# Playing Exergames Facilitates Central Drive to the Ankle Dorsiflexors During Gait in Older Adults; a Quasi-Experimental Investigation

**DOI:** 10.3389/fnagi.2019.00263

**Published:** 2019-09-20

**Authors:** Eling D. de Bruin, Nadine Patt, Lisa Ringli, Federico Gennaro

**Affiliations:** ^1^Institute of Human Movement Sciences and Sport, Department of Health Sciences and Technology, ETH Zurich, Zurich, Switzerland; ^2^Division of Physiotherapy, Department of Neurobiology, Care Sciences and Society, Karolinska Institutet, Stockholm, Sweden; ^3^SRH Hochschule für Gesundheit, Gera, Germany

**Keywords:** older adults, exergame, central drive, tibialis anterior, foot clearance

## Abstract

**Purpose:**

Gait training might be of particular importance to reduce fall risk in older adults. In the present study we explore the hypothesis that video game-based training will increase tibialis anterior (TA) muscle EMG-EMG coherence and relates to functional measures of lower limb control.

**Methods:**

We focus on video game-based training performed in standing position, where the subjects have to lift their toes to place their feet on different target zones in order to successfully play the game. This type of training is hypothesized leading to progressive changes in the central motor drive to TA motor neurons and, consequently, improved control of ankle dorsiflexion during gait.

**Results:**

Twenty older adults, 79 ± 8 years old, 13 females/7 males, participated. Results showed a significant difference against 0 in the experimental ΔPOST condition in dual-task walking and beta Frequency Of Interest (*p* = 0.002). Walking under dual task condition showed significant change over time in minimal Toe Clearance for both the left [χ^2^(2) = 7.46, *p* = 0.024, *n* = 20] and right [χ^2^(2) = 8.87, *p* = 0.012, *n* = 20] leg. No change in lower extremity function was detectable.

**Conclusion:**

Overall we conclude that the initiation of an exergame-based training in upright standing position improves neural drive to the lower extremities in older adults, effects on minimal Toe Clearance and seems an acceptable form of physical exercise for this group.

## Introduction

Increasing the number of years of good health while maintaining independence and quality of life as long as possible is a primary public health goal. Avoidance of disease and disability, maintaining high physical and cognitive function, and sustained engagement in social and productive activities are components of healthy aging that together define *successful aging* (Rowe and Kahn, 1997).

A large component of successful aging interventions aims to maximize physical performance levels. Being able to fully participate in daily life activities may be affected when the capability to easily perform common physical functions decreases (Rowe and Kahn, 1997). Consequently, in older adults health status can be regarded an important indicator of quality of life (Johnson and Wolinsky, 1993; Spirduso and Cronin, 2001). The way how middle-aged and older adults perceive their health seems especially related to components of health-related fitness and functional performance, or to chronic conditions and diseases that influence these fitness components (Johnson and Wolinsky, 1993; Malmberg et al., 2002; Malmberg et al., 2005).

Older adults that are physically active or who regularly exercise help in preventing the development and progression of chronic degenerative diseases (Physical Activity Fundamental To Preventing Disease, 2002; Chodzko-Zajko et al., 2009; Stuart et al., 2009). Being physically active or adherent to regular exercise are means to consistently improve age-related muscle weakness, physical function, cognitive performance, and mood in older adults (Landi et al., 2010). Where a sedentary lifestyle in older adults will increase the risk of unintentional falls, it is observable that falls risk is reduced by being physically active (Thibaud et al., 2012). Approximately 30% of older people experience falls on a yearly basis (Berg et al., 1997; Hausdorff et al., 2001; Gill et al., 2005) and falling can be seen as a common problem in the growing elderly population (de Bruin et al., 2012).

Individuals with gait impairments have an increased risk for repeated falls (Tinetti et al., 1995) and, accordingly, walking ability and the risk of falling are linked with each other. The three most frequent motor control related direct causes of falling are tripping, slipping and loss of balance (Lord et al., 1993; Sherrington et al., 2004). Furthermore, forward walking activity is associated with a high proportion of falls (Robinovitch et al., 2013).

Greater variability in Minimum Foot Clearance (MFC) is a contributing factor to a trips risk increase, and concomitant associated heightened falls occurrence, in those cases where older are compared with younger adults and older fallers to older non-fallers. MFC is “the minimum vertical distance between the lowest point of the foot of the swing leg and the walking surface during the swing phase of the gait cycle (Barrett et al., 2010).” Toe clearance control with the central nervous system in critical situations is impaired in older adults at high risk of tripping (Hamacher and Schega, 2014), an observation in line with research indicating that corticospinal transmission to skeletal muscle may be impaired with advancing age (Manini et al., 2013).

Motor-cognitive training with exergames may help in effectively supporting physical, psychological, and cognitive rehabilitative outcomes in older adult populations (Zeng et al., 2017; van Santen et al., 2018). Patients with mobility problems, for example, have shown transfer of training effects obtained in a virtual environment to real-life (Erren-Wolters et al., 2007). Two systematic reviews have shown that especially cognitive-motor stepping interventions with video-games positively effect on gait of older adults (Schoene et al., 2014; Okubo et al., 2017) and this approach, furthermore, is considered task-specific for neuroplasticity improvements (Netz, 2019). Previous studies from our group pointed to the potential of video game-based training and highlighted that specifically designed game-based training improves walking (Pichierri et al., 2012a, b; Fraser et al., 2014) and effects on the brain (Eggenberger et al., 2016; Schattin et al., 2016; Stanmore et al., 2017). The game play also requires ankle dorsiflexion to play the game, a MFC-associated factor (Sato, 2015). Here we explore the hypothesis that video game-based training effects on corticospinal transmission to the tibialis anterior (TA) muscle assessed by means of EMG-EMG coherence and lower limb control. We focus on video game-based training performed in standing position, where the subjects have to lift their toes to place their feet on different target zones in order to successfully play the game. We hypothesize that this type of training leads to changes in the central drive to TA and, thus, to improved ankle dorsiflexion control during gait.

## Materials and Methods

In this “pretest – posttest” quasi experimental single group design the older adults acted as their own controls to control for inter-subject variability (Willerslev-Olsen et al., 2015). Comparing against a not training group of older adults would complicate the comparison of data given the expected diversity of functional abilities in older adults. Furthermore, this design allowed determining whether intervention effects can be explained by simple test-retest variability and whether significant changes would be observed in the measured variables during a 6-week control period comparable to that of the 6-week intervention period.

The study was designed for autonomous and independent living older adults. For study inclusion, the older adults had to be 65 years or above, had to be in good physical health by self-report (assessed by means of a Health Questionnaire), had to be able to walk at least 500 m independently (with or without walking aids), and they had to be not experienced in exercising with virtual reality-based games before this study. Moreover, they were considered eligible if they had not been diagnosed for cognitive impairments and they had a Montreal Cognitive Assessment (MoCA) score of 26 or more points. Participants were not eligible when exhibiting acute or unstable chronic diseases, rapidly progressing or terminal illnesses or were suffering from severe health problems (e.g., recent cardiac infarction, uncontrolled diabetes or hypertension). The ethics committee of the ETH Zurich, Switzerland (EK 2017-N-22) approved the study protocol. Before any measurements were performed, an informed consent according to the Declaration of Helsinki was administered and signed by each eligible participant.

### Protocol and Training Intervention

Twenty older adults (average age 79 years old, 13 females, and 7 males) from the Alterszentrum Kehl (Baden, Switzerland) were recruited and participated voluntarily during a 12 weeks period. Gait analysis, EMG recordings of TA activity, physical functioning, and cognition were assessed during three test sessions separated by approximately 6 weeks intervals. All test sessions took place in the Alterszentrum Kehl (Baden, Switzerland^1^). The first test session occurred approximately 6 weeks before training commenced, and the second testing was organized 1 week before training initiation. The final test session took place following the last training day. All three testing sessions included the same measurements performed in the same order on every occasion and at the same time of the day. Test sessions began with concurrent gait analysis and EMG recordings during over ground walking, followed by lower extremity function and cognitive testing.

**FIGURE 1:**
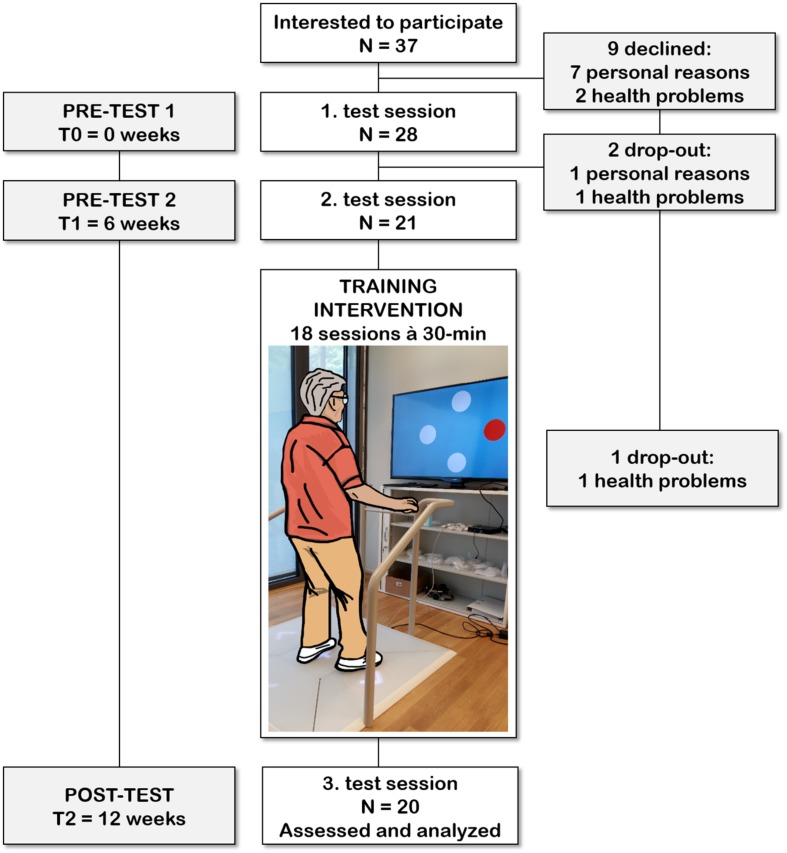
Participant flow chart with an exemplified use of the exergame platform used for this study. Each participant was involved in the study for a period of 12 weeks. After the 6 weeks control period (between the 1 and 2 test session), each participant trained 18 times within a period of 6–7 weeks.

The participants performed a total of 18 training sessions lasting 20 min (with a break of 10 min in between). Each training session was performed on a Senso exergame system (dividat, Schindellegi, Switzerland; Figure 1). The Exergame had to be played using body movements to trigger sensors positioned on a base plate that was connected to a TV screen. Through these body movements participants apply forces and steps of which the dynamics are recorded. Real-time visual and auditory feedback on Exergame performance was provided through electronic sensors in the dance pad detecting position and timing information.

The equipment was set up in a quiet room in the Alterszentrum Kehl (Baden, Switzerland). The participant stood in front of a computer screen positioned at eye-level during training. A handrail was placed in front of the participant to provide safety support, if needed. During the first session exercise tasks were explained by a study coordinator to the participant. During subsequent sessions, participants conducted training using screen-based feedback only. To guarantee safety the study coordinator remained with the participant during training.

The used exergames are designed to train different cognitive domains; e.g., divided attention, working memory, inhibition, attention shifting, spatial orientation and postural control (Table 1). The training intervention program was specifically designed for healthy seniors and the participants were gradually accustomed to the games. During the individual training period, each participant was constantly monitored by the instructors and received one-on-one supervision. The training protocol contained a variety of exercises in which the difficulty progressively adapted to an individual skill level. Attendance to the training sessions was monitored by the instructors. Participants were provided with individual diaries at each training session, to record the date of training, which specific exergames were used, and both intensity and duration of each exergame performed in that session.

**TABLE 1:** Description of the video games.

**Video game**	**Description**	**Domain**
Simple	There are four white circles on the screen. If one of the four circles appears red, the participant has to react as fast as possible and therefore to make a step in the appropriate direction to push the arrow.	Attention and processing speed
Targets	There are four targets on the screen and flying balls appear on the screen from all sides. As soon as one of the balls crosses the center of one of the targets, the participant has to make a step in the appropriate direction to push the arrow. To reach the maximal score, the arrow has to be pushed exactly at the time when the ball is in the center of the target.	Reaction time and precision
Divided	The participant has to focus on visual and auditory stimuli. This game is an extension of the game “Simple”. Participants have not only to react to the circles becoming red but also to auditory stimuli. If a high tone appears, the participants have to make a step forward, if a low tone appears, participants have to make a step backward.	Divided attention
Simon	Various tones are played and represented spatially on the screen. The participant has to memorize the sequence and reproduce it by pushing the appropriate arrows.	Working memory
Flexi A+B	A: There’s a number in the middle of the screen. Around this number are four other numbers. The arrow with the next higher number according to the number in the middle has to be pushed.	Cognitive flexibility
	B: Additionally, a pattern appears around the number. Now the arrow with the next higher number and the opposite pattern has to be pushed.	
Snake	A white snake is navigated on the screen through the participant stepping on the appropriate arrows on the plate. The snake has to eat the red squares.	Visuo-Spatial processing
Seasons	Participants have to react and simultaneously inhibit reaction to stimuli appearing on the screen. The screen is divided into four seasons. There appear objects which either fit to the season or not. Participants have to push the appropriate arrow if the objects do not fit into the season.	Selective attention and inhibition
Tetris	There are blocks falling down slowly from the upper end of the screen. Participants have to move the blocks to build rows on the lower end of the screen, preferably without gaps. A row disappears as soon as one is complete. With the arrow in front, participants can turn the block by 90 degrees. With the left and right arrows the block can be moved into the according direction. With the arrow behind, the block goes faster to the bottom of the screen.	Spatial orientation

### EMG Data Acquisition and Pre-processing

**FIGURE 2:**
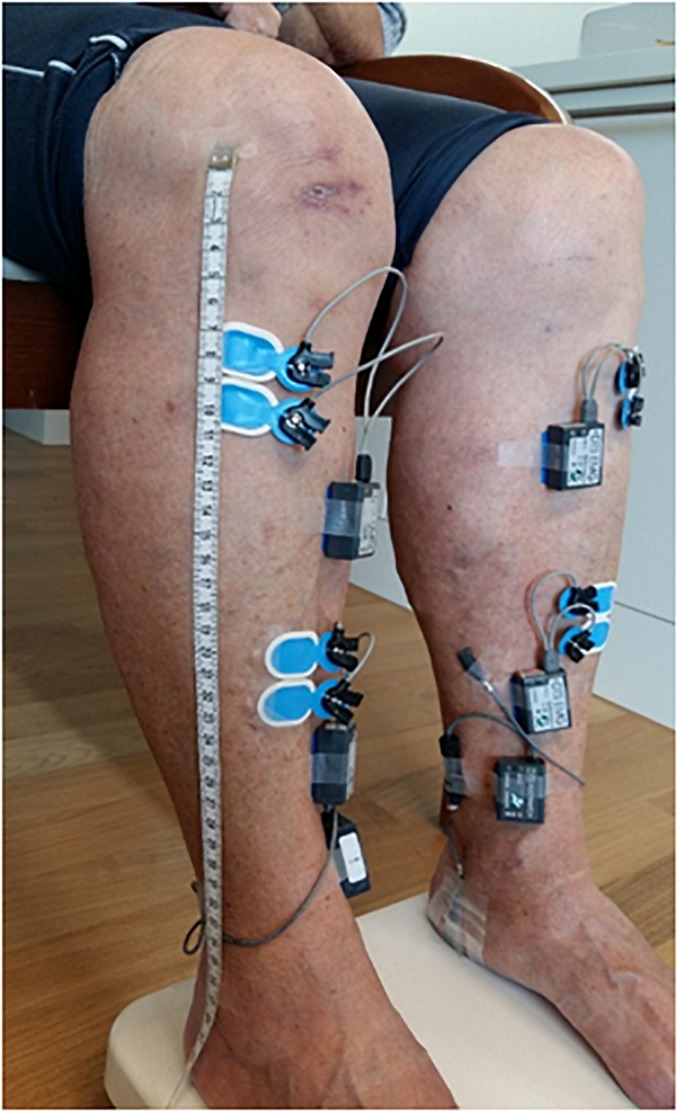
Placement over both tibialis anterior muscles of a pair of bipolar EMG electrodes using an inter-electrodes distance of ∼2 cm in each bipolar set and a distance of ∼10 cm between the two bipolar set of EMG electrodes to reduce the risk of cross-talk. Footswitches placed over the midpoint of each heel were placed in order to record heel-strike events during the overground gait trials.

Surface EMG signals were recorded at a sampling frequency of 1500 Hz (Noraxon DTS TeleMyo, Scottsdale, AZ, United States). For this two pairs of bipolar Ag-AgCl electrodes (Ambu Blue Sensor N, Ambu A/S, Ballerup, Denmark) over the left and right TA muscles (Figure 2) were used. Each bipolar configuration of the pair was placed either proximally or distally with respect to the muscle belly according to previously described anatomical landmarks (van Asseldonk et al., 2014). The inter-electrodes distance (electrodes’ center-to-center) was set to 2 cm, whereas the two bipolar configurations, within each pair, were separated by ∼10 cm (range: ∼8.50 cm to ∼13.50 cm) in order to reduce the risk of cross-talk as well as the recording of muscle activity from overlapping motor unit areas (Hansen et al., 2005). To ensure the replication of the two bipolar placements across all the assessments, placements (for each TA muscle) were recorded by keeping trace of distance metrics from anatomical landmarks at each subject level. The skin was prepared (i.e., cleaned and, when necessary, shaved) before placing the EMG electrodes using a specific paste (H+H Medizinprodukte GbR, Münster, Germany). Moreover, in order to detect the onset of the heel strikes, two footswitches were placed approximately on the midpoint of the calcaneus in both feet. Continuous EMG data was first demeaned and de-trended by removing the zero- and first-order polynomial, respectively, and then high-pass filtered at 10 Hz (zero-phase 4th-order Butterworth filter). Subsequently, powerline noise and its harmonics were filtered out using a notch filter based on Discrete Fourier Transformation (DFT) followed by a low-pass filter at 500 Hz (zero-phase 4th-order Butterworth filter) and rectification of the Hilbert transform of the filtered data. The latter non-linear transformation produces an output similar to performing solely rectification without previous transforming by Hilbert (Myers et al., 2003; Boonstra and Breakspear, 2012). This approach represents a widely used strategy in the preprocessing steps before computing either cortico-muscular coherence (i.e., by means of EEG and EMG) or intramuscular coherence (Schoffelen et al., 2011; Boonstra et al., 2015). Considering the debate on rectification as a proper preprocessing step before coherence analysis (Halliday and Farmer, 2010; Neto and Christou, 2010), the adopted filtering strategy with included rectification has shown to produce more reliable EMG-EMG coherence results during walking in comparison to different preprocessing strategies (van Asseldonk et al., 2014). The left and right heel-strike events were merged and used to epoch the EMG data relative to the gait cycle, similar as in previous analyses (Petersen et al., 2012; van Asseldonk et al., 2014; Willerslev-Olsen et al., 2015; Kitatani et al., 2016). The duration of the resulting epochs started one sample before the onset of the heel-strike (to avoid excessive contamination with artifacts of the EMG data caused by the collision of the foot with the ground) to the preceding 400 ms. The preprocessed EMG data segments were subsequently down-sampled to 500 Hz.

### Spectral Analysis of EMG Data

A multi taper frequency transform was used to achieve cross- and power spectra from the preprocessed data segments, by tapering each of these with a variable set of discrete prolate spheroidal (Slepian) sequences. The used epochs of data of 400 ms duration, yielded to a frequency resolution of 2.5 Hz. A broad power- and cross-spectra was calculated (0–100 Hz). Within these, three frequency bands were of interest (FOI) for further analysis: beta (15–30 Hz), low-gamma (32.5–47.5 Hz), and high-gamma (50–65 Hz). The focus on the first two FOI (beta and low-gamma) is based on mounting evidence of a high association of the coherence in these frequency bands (gathered range: ∼15–50 Hz) with the quality of the neural drive to the muscle during walking and, therefore, with the integrity of the pyramidal system. Furthermore, they also show a high potential of being modifiable by several types of training intervention; e.g., gait training (Norton and Gorassini, 2006; Barthelemy et al., 2010; Petersen et al., 2012; Willerslev-Olsen et al., 2015; Kitatani et al., 2016). The focus on the third FOI (high-gamma) is based on its high association with corticospinal interaction effectiveness, as shown in experimental paradigms involving both motor and cognitive resources; e.g., reaction time readiness (Schoffelen et al., 2005), where the role of coherence in high-gamma during locomotion (e.g., gait) has been investigated in first promising research work (Clark et al., 2013).

Two sets of tapers were adopted in two runs, to obtain an optimal spectral concentration and sensitivity relative to the three FOI. For the FOI up to 30 Hz (i.e., beta) three tapers were used with a resulting spectral smoothing of ±5 Hz around each frequency bin, whereas for the FOI higher than 30 Hz (i.e., low- and high-gamma) nine tapers were used with a resulting spectral smoothing of ±12.5 Hz around each frequency bin. Usually, the beta frequency bandwidth is represented by ∼10 Hz and the gamma frequency bandwidth by ∼25 Hz (Schoffelen et al., 2011), thus supporting the utilization of this spectral smoothing strategy. The following equation was used to calculate power- and cross-spectra:

(1) $$S_{x\,y}\,\left(f\right)=F_{x}\,\left(f\right)\times F_{y}\,{\left(f\right)}^{{}^{*}}$$

where *F*_*x*_(*f*) [or *F*_*x*_(*f*)] denotes the Fourier transform of the signal *x* (or *y*) relative to the frequency *f* and ^∗^ denotes the complex conjugate. In this analysis signal *x* and signal *y* represent proximal and distal EMG data segments, respectively. When *x*≠*y*, *S*_*xy*_(*f*) denotes the cross-spectra between signal *x* and signal *y*, relative to the frequency *f*. When *x=y*, *S*_*xy*_(*f*) is reduced to *S*_*xx*_(*f*) [or *S*_*yy*_(*f*)], which consists of the (auto) power spectra of the signal *x* (or *y*), relative to the frequency *f*. Single segments of power- and cross-spectra yielded after averaging across tapers were used in order to calculate the coherence estimate between proximal and distal EMG data, with the following equation:

(2) $$C\,o\,h_{x\,y}=\frac{\left|\text{⟨}\,S_{x\,y}\,\text{⟩}\right|}{\sqrt{\text{⟨}\,S_{x\,x}\,\text{⟩}\times \text{⟨}\,S_{y\,y}}\,\text{⟩}}$$

where ⟨⋅⟩ denotes the obtained power- or cross-spectra after averaging across data segments. Coherence is a spectral measure representing the linear correlation between signal *x* and signal *y*, where the estimate ranges between 0 and 1, with 0 representing no linear association and 1 perfect relation at a specific frequency *f*.

In previous studies, 70–100 heel-strikes (i.e., data segments) were used to calculate the coherence estimates (Norton and Gorassini, 2006; van Asseldonk et al., 2014). However, it has been shown that a rather small number of trials (i.e., 25 or 50) may result in a larger variability of gait-related coherence estimates, which tends to decrease as the number of segments increase (van Asseldonk et al., 2014). Notably, a large number of trials (i.e., 200) has been shown to relate with a still rather large variability (i.e., 50%) to gait-related coherence estimates (van Asseldonk et al., 2014). This large variability may be problematic when comparing coherence estimates obtained from an unequal number of segments within several testing sessions (Maris et al., 2007). In this study, all the data segments from the obtained heel-strike events in all the measurement time points were used for further analysis (mean ± SD across the three measurement time points in normal and dual-task walking: 224 ± 96, 213 ± 102, 246 ± 89, 244 ± 106, 212 ± 102, 252 ± 80, respectively).

### Gait Analysis

Toe clearance (cm), was measured with the Physilog (Gait up Sàrl, Lausanne, Switzerland) wearable movement sensors (50 × 37 × 9.2 mm, 19 g). Data transfer to a computer for further analysis was allowed through a micro-USB port. Elastic straps fixed the sensors at the right and left forefoot of the participants to allow flat over ground gait analysis. Physilog provides a valid quantitative assessment of gait kinematic parameters (Aminian et al., 1999; Dubost et al., 2006; de Bruin et al., 2007).

**FIGURE 3:**
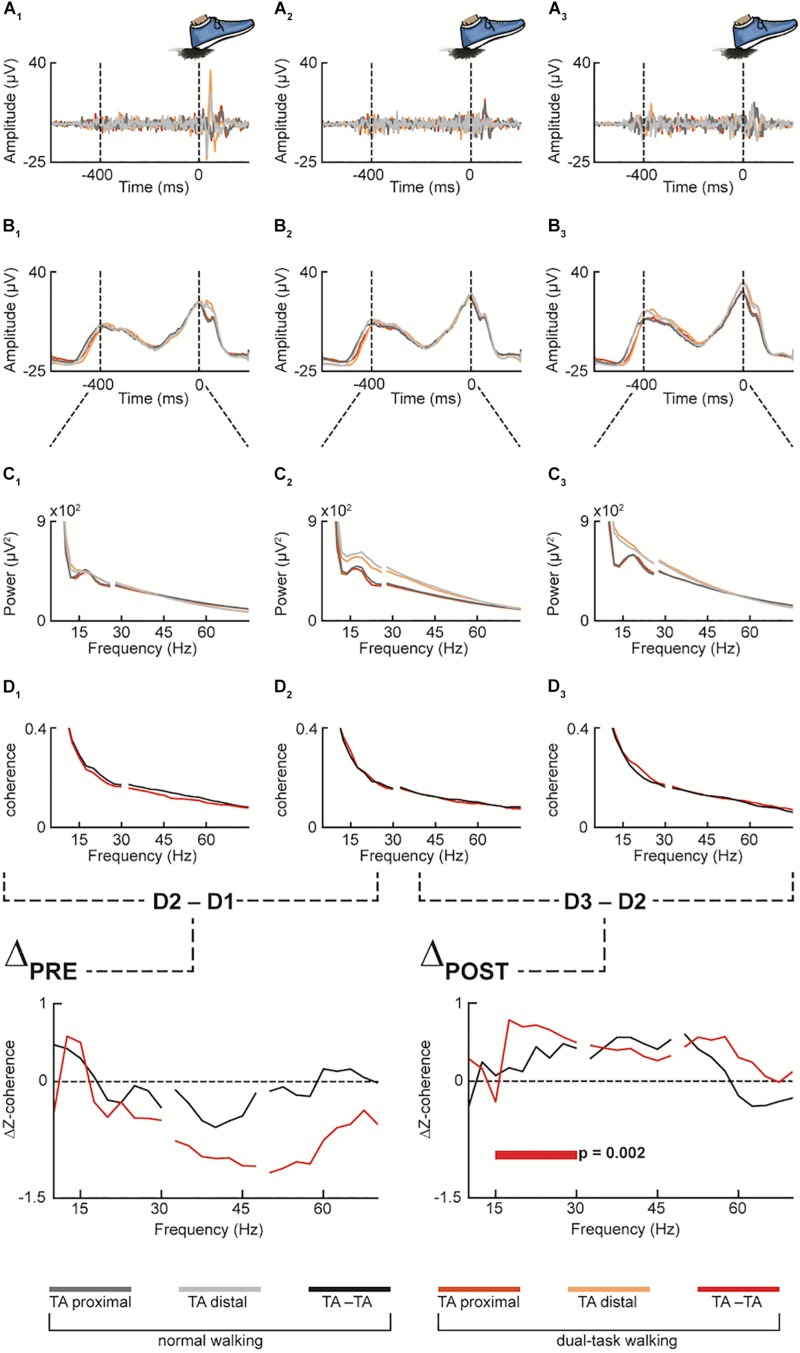
Pooled data for both normal and dual-task walking (in grayish and reddish color palette, respectively), across the three measurement time points (1st pre-intervention, 2nd pre-intervention and post-intervention, denoted by the numeric subscripts: 1, 2 and 3, respectively) at different preprocessing stages: Proximal and distal emg sensors over the tibialis anterior muscle (TA) were first filtered **(A1–A3)** and then rectified **(B1–B3)**. The filtered and rectified emg data were then epoched from the heel strike (excluded) to the preceding 400 ms before performing spectral analysis of frequency **(C1–C3)** and intramuscular coherence **(D1–D3)** between proximal and distal TA (TA-TA). Then, Z-transformed coherence of the 1st pre-intervention measurement **(D1)** was subtracted from the Z-transformed coherence of the 2nd pre-intervention measurement **(D2)** as well as **(D2)** from the post-intervention measurement **(D3)** (ΔPRE and ΔPOST, respectively). In the Dual-Task condition (red color) of ΔPOST, statistically significant difference of Δz-coherence resulted in the averaged beta frequency band of the spectrum (i.e., 15–30 Hz).

A figure-8 walking path was settled up, by placing two structures at a distance of approximately 7 m, and a gait testing protocol with at least 50 gait cycles (five repetitions of the path; Figure 3) had to be accomplished by the participants. Such a number of gait cycles has been shown to be sufficient to reliably estimate gait kinematics parameters (König et al., 2014). Participants performed five repetitions of the figure-8 gait path during both single- and dual-task condition as well as both under preferred and fast walking speed. In the dual-task walking condition, participants counted backward in steps of seven after receiving a random given number between 200 and 250. The participants were told to count loud; otherwise, the trial was recorded as failure. The dual task-condition quantifies the automaticity of movement and multi-tasking capabilities (Abernethy, 1988; Wright and Kemp, 1992; Wulf et al., 2001) and assessed the control of MTC under dual task conditions as a marker of motor control (Hamacher et al., 2016). For each participant, we calculated the variability of toe clearance (Mills et al., 2008). A measure of variability (e.g., standard deviation) of MTC height in preferred walking is indicative for diminished gait control in older adults (Begg et al., 2007; Mills et al., 2008).

### Lower Extremity Functioning

The Short Physical Performance Battery (SPPB) was used to assess lower extremity functioning. The test battery contains (i) a balance test, (ii) a 4-meter gait test, and (iii) a 5-chair-rise test. Timed results from each test are categorized into variables ranging from 0 (unable to perform) to 4 (best performers) according to well-established cut-off values (Guralnik et al., 2000). The sum of the results [tests (i)–(iii); theoretically ranging from 0 to 12] is used for the analyses where 12 indicates the highest degree of functioning.

At the pre-clinical stage this test is a predictor of subsequent disability (Vasunilashorn et al., 2009) and is applicable in routine clinical settings for monitoring of functioning (Perera et al., 2005). Test administration criteria have been published at “www.grc.nia.nih.gov/branches/ledb/sppb/index.htm.” The participants were tested within a single session lasting around 10 min.

### Montreal Cognitive Assessment

The MoCA “paper-and-pencil-test” screens cognitive domains such as memory, language, executive functions, visuospatial skills, attention, concentration, and orientation (Nasreddine et al., 2005; Julayanont et al., 2013). The maximal score possible is 30 points and reflects a quantitative estimate of the overall cognitive abilities (Koski et al., 2011). A cut-off score below 26 points is often taken as indicator of possible mild cognitive impairment and dementia. The instrument is sensitive to change (Tiffin-Richards et al., 2014) and available in German.

### Statistical Analysis; EMG-EMG Coherence

Statistical analysis of the EMG-EMG coherence estimates was performed as follows. The Z-coherence spectra z(*f*) was computed at subject level to account for the variability before performing statistical testing according previously reported procedures (Maris et al., 2007; Schoffelen et al., 2011) and calculating the estimated change between measurement time points, using:

(3) $$Z\,\left(f\right)=\;\frac{\left(T\,a\,n\,h^{-1}\,\left(C\,o\,h_{1}\right)-1/\left(2\,t\,a\,p_{1}-2\right)\right)-\left(T\,a\,n\,h^{-1}\,\left(C\,o\,h_{2}\right)-1/\left(2\,t\,a\,p_{2}-2\right)\right)}{\sqrt{(1/\left(2tap_{1}-2\right)+1/\left(2tap_{2}-2\right)})}$$

where *Tanh*^−1^ is the inverse hyperbolic tangent, *Coh*_*1*_ denotes the coherence estimate in measurement time point *n* and *tap*_*n*_ denotes the total number of tapers used for the spectral estimation of the frequency in measurement time point *n*. The total number of tapers (*tap*_*n*_) was obtained by multiplying the tapers used (in this analysis either 3 or 9, as described above) by the number of data segments for each subject and measurement time point. The individual ΔZ-coherence spectra obtained using equation (3) were performed by subtracting, for each frequency band, the 1st measurement time point from the 2nd (Δ_*PRE*_) and the 2nd measurement time points from the 3rd (Δ_*POST*_). For the study design here presented, the Δ_*PRE*_ was considered the “control” condition while Δ_*POST*_ represented the “experimental” condition. This is assuming that no significant difference is expected from pre-measurement time points one to two, and a significant difference is expected between the 2nd and the 3rd measurement time points.

Statistical inference was based on pooled individual Δ_*PRE*_ and Δ_*POST*_ separately for each using a non-parametric permutation test approach as described elsewhere (Maris et al., 2007; Schoffelen et al., 2011). Briefly, pooled Δ_*PRE*_ and Δ_*POST*_ for both normal and dual-task walking condition and for each frequency band (where the average across frequencies, within frequency band, was used) were tested for significant differences from 0 by approximating the *p*-values by 100000 Monte Carlo permutations. The level of significance was set to α = 0.05 and a two-tailed test was adopted and corrected by multiplying the *p*-values with a factor of two prior to α thresholding.

### Statistical Analysis; Secondary Outcomes

Normality was tested for the remaining outcomes data before analysis with the Shapiro–Wilk test. Then the Friedman test was used to test for differences between time points because of non-normal data distributions. *Post hoc* analysis with Wilcoxon signed-rank tests and Dunn-Bonferroni correction was conducted when the Friedman test revealed significant values. All values are reported as means ± 95% confidence intervals (CI) and all statistical analyses were performed with SPSS.

Pearson’s correlation was used for Effect Size determination with *r* = 0.1 meaning “small”, *r* = 0.3 “medium,” and *r* = 0.5 meaning a “large” effect (Cohen, 1988).

## Results

Demographics and clinical characteristics of included participants (*N* = 20) is summarized in Table 2.

**TABLE 2:** Demographics and clinical characteristics of participants (*N* = 20).

**Demographic characteristics**		
Gender (female/male)	13/7	
Age in years (mean ± SD)	79 ± 8	
BMI in kg/m^2^ (mean ± SD)	25.37 ± 5.42	
MoCA (mean ± SD)	26.45 ± 1.877	
SPPB (mean ± SD)	10.40 ± 1.667	
Training Compliance (%)	100	

**Walking aids**	***n***	**%**

Stick	1	5
Walker Rollator	0	0
Diabetes mellitus	2	10
Polyneuropathy	0	0
Hypertonia	11	55
Cardiac insufficiency	1	5
Cardiac infarction	1	5
Stroke	3	15
Cancer	1	5
Respiratory diseases	0	0
Gastro-intestinal diseases	1	5
Arthropathy	9	45
Osteoporosis	7	35
Eye diseases	9	45
No diseases	1	5
**Prescription medications**		
0–3	11	55
3–6	6	30
>6	3	15
**Falls in last 6 months**		
No falls	13	65
1 fall	6	30
>1 fall	1	5

*SD = standard deviation; *n* = number of participants.*

### EMG-EMG Coherence

All the control ΔPRE conditions revealed non-significant differences in both normal (*p* = 0.815, *p* = 0.428, and *p* = 0.963, for beta, low and high-gamma FOI, respectively) and dual-task walking (*p* = 0.503, *p* = 0.056, and *p* = 0.066, for beta, low and high-gamma FOI, respectively).

Regarding the coherence estimates during normal and dual-task over ground walking, the non-parametric permutation test showed a significant difference against 0 in the experimental ΔPOST condition in dual-task walking and beta FOI (*p* = 0.002), while no significant difference was observed in the low and high gamma FOI (*p* = 0.307 and *p* = 0.372, respectively). No significant difference was observed in the normal walking condition in both beta, low and high gamma FOIs (*p* = 0.168, *p* = 0.257, and *p* = 0.715, respectively).

### Gait Analysis and MTC

No significant change over time for MTC was observed for walking under single task condition.

Walking under dual task condition showed significant change over time in MTC for both the left [χ^2^(2) = 7.46, *p* = 0.024, *n* = 20] and right [χ^2^(2) = 8.87, *p* = 0.012, *n* = 20] leg.

*Post hoc* testing (Dunn-Bonferroni) revealed the difference (left leg) to be between time points 1 and 3: *p*_1__–__3_ = 0.027; *p*_1__–__2_ ≥ 0.9; and *p*_2__–__3_ = 0.173. Effect sizes were large and medium-large (time point 1 – 3 *r*_1__–__3_ = 0.58 and time point 2 – 3 *r*_2__–__3_ = 0.42). A small-medium Effect resulted for time point 1 – 2 (*r*_1__–__2_ = 0.16).

*Post hoc* testing (Dunn-Bonferroni) revealed the difference (right leg) to be between time points 1 and 2: *p*_1__–__2_ = 0.022; *p*_1__–__3_ ≥ 0.9, *p*_2__–__3_ = 0.066). Effect sizes were large (time point 1 – 2 *r*_1__–__2_ = 0.6 and time point 2 – 3 *r*_2__–__3_ = 0.51) and small (time point 1 – 3 *r*_1__–__3_ = 0.08).

### Secondary Outcomes

The inferential statistical testing for the secondary outcomes, revealed no significant changes in lower extremity functioning: χ^2^(2) = 3.68, *p* = 0.159, *n* = 20, whereas a significant change in MoCA scores over time was observed with χ^2^(2) = 11.76, *p* = 0.003, *n* = 20). Pairwise comparisons revealed this difference to be between time points 1 and 3 (*p*_1__–__3_ = 0.008; *p*_1__–__2_ = 0.081; and *p*_2__–__3_ ≥ 0.9) (Table 3).

**TABLE 3:** Resulting MTC, SPPB, and MoCA values at the different measurement time points.

		**TP 1**	**TP 2**	**TP 3**	***P-value***
		**Mean ± SD**	**Mean ± SD**	**Mean ± SD**	
**ST**					
MTC (m)	*Left*	0.028 (0.01)	0.026 (0.01)	0.026 (0.009)	0.267
	*Right*	0.023 (0.01)	0.025 (0.01)	0.024 (0.008)	0.071
**DT**					
MTC (m)	*Left*	0.029 (0.009)	0.027 (0.009)	0.025 (0.009)	0.024
	*Right*	0.021 (0.009)	0.024 (0.01)	0.022 (0.008)	0.012
SPPB		11 (3)	10.5 (2)	11 (1)	0.159
MoCA		27 (3)	28 (3)	28.5 (3)	0.003

*TP = time point; ST = single task; DT = dual task; min = minimum toe clearance; SD = standard deviation.*

The amount of points achieved in the video games in the final training week was significantly higher compared to the 1st week of training for all video games played (“Simple”: *z* = −3.659, *p* < 0.001; “Targets”: *z* = −3.920, *p* < 0.001; “Divided”: *z* = −3.920, *p* < 0.001; “Simon”: *z* = −3.884, *p* < 0.001; “Flexi A+B”: *z* = −3.921, *p* < 0.001; “Snake”: *z* = −3.921, *p* < 0.001; “Tetris”: *z* = −3.809, *p* < 0.001) (Table 4).

**TABLE 4:** Points achieved in the video games in the 1st week vs. the last week.

**Week 6 – Week 1**	***z***	***p* (two-tailed)**
**Game**		
Simple	−3.659	<0.001
Targets	−3.920	<0.001
Divided	−3.920	<0.001
Simon	−3.884	<0.001
Flexi A+B	−3.921	<0.001
Snake	−3.921	<0.001
Tetris	−3.809	<0.001

*The points from seven games were used for the analysis. It was not possible to extract the points from the game “Seasons.” *n* = 20 participants completed all 18 training sessions and were used for the analysis in this table.*

## Discussion

This study aimed to explore whether video game-based training effects on the central drive to the ankle dorsiflexors during over ground walking and on MTC as measure of lower limb control. We hypothesized that this type of training would lead to changes in the corticospinal transmission to TA muscles and, thus, to improved motor control of ankle dorsiflexion while walking. The main finding was an observed change in the experimental ΔPOST condition in dual-task walking and the beta FOI which, thus, indicates the intervention effected on neural drive through enhanced quality of the neural drive (Norton and Gorassini, 2006; Barthelemy et al., 2010). This finding closely resembles investigations where intramuscular coherence in the beta frequency has shown to be related to neural drive to the muscle and, thus, the control of gait (Petersen et al., 2012; van Asseldonk et al., 2014; Kitatani et al., 2016). Furthermore, this finding gets supported by the observed change of the mean value of MTC that is accompanied by decreasing standard deviation values. Evidence of a possible linkage between lower extremity activation through training and changes in the neural drive also stem from a study from Dalgas et al. (2013). These authors showed that lower extremity resistance training in multiple sclerosis improves the neural drive to lower limb muscles. Similar results for resistance training for non-impaired individuals were reported (Aagaard et al., 2002). However, changes in the neural drive are also seen with other forms of initiating chronic physical activity (Enoka, 1997). This study is the first to show enhanced neural drive due to an exergame intervention. This finding is relevant for our society that is currently challenged to find an answer for supporting public health policies aimed at helping senior citizens achieving the goals of primary and secondary (i.e., reducing readmission rates) prevention to remain independence in functioning (McCaskey et al., 2018). Physical activity and exercise for older adults may help in this context to foster physical and cognitive functioning at the highest possible levels (DiPietro, 2001; American College of Sports Medicine, 2004; Elsawy and Higgins, 2010; de Souto Barreto et al., 2016; Karssemeijer et al., 2017) and considering that motivation to continue and adhere to conventional exercise is often difficult (Phillips et al., 2004). More research that aims at establishing the best ways to encourage older adults to be more physically active in the long term is needed (Bennett and Winters-Stone, 2011). The incorporation of progressively intense but short exercise as part of a tailored and combinatory program; e.g., with exergames, may be beneficial (Hwang et al., 2016). This type of training is also feasible for co-morbid geriatric patients Guadalupe-Grau et al. (2017).

The exergame used in this study is a motor-cognitive exercise from which can be hypothesized that it improves the synapse communication in brain networks responsible for movement coordination and execution (Eggenberger et al., 2016; Schattin et al., 2016; McCaskey et al., 2018). This, in turn, could positively influence the communication from the motor area of the brain to the muscles. Previous studies pointed to the potential of video game-based training and highlighted that video games that are specifically designed for training purposes improve walking (Pichierri et al., 2012a, b; Fraser et al., 2014) and effect on the brain (Eggenberger et al., 2016; Schattin et al., 2016). Emerging evidence indicates that part of the age-associated loss in muscle strength is due to an impaired communication between the brain and the effector organs (Clark and Manini, 2008; Manini et al., 2013); e.g., the muscles of the lower extremities. Furthermore, such deficits in the neural drive can lead to much of the muscle weakness observed in older adult populations (Clark and Taylor, 2011). Improved neural drive to the lower extremity muscles might explain why strength gains in lower extremity muscles are observable following exergame interventions (Jorgensen et al., 2013; Sato et al., 2015).

Older adults exhibit large muscle activation deficits (Clark and Fielding, 2012) which may explain the divergence between the rates in loss of strength and muscle mass, and may account for up to one third of loss in force production. Furthermore, traditional rehabilitation programs that use conventional strength training in patients with impaired muscle activation may not be optimal to reverse the loss in muscle strength (Stevens et al., 2003). Part of the muscle strength loss seen in older adults may be due to qualitative and quantitative changes in the motor cortex that negatively impacts on voluntary activation of the muscles (Clark and Taylor, 2011). Voluntary activation can been defined as the “level of voluntary drive during an effort” (Gandevia, 2001; Taylor, 2009). The descending drive from the motor cortex is considered the major determinant of the timing and strength of voluntary contractions (Clark and Taylor, 2011). Motor unit firing rates for the ankle dorsiflexor, for example, show slower rates for older than younger men (Roos et al., 1997; Todd et al., 2003). It can be hypothesized that training programs that focus on aspects of voluntary muscle activation in addition to more conventional types of resistance training, such as in the exergame we used, may result in greater strength gains in individuals that show larger voluntary activation deficits. Future longitudinal studies that use such combinations are warranted.

Our result seems at variance with the lack of an effect on the lower extremity function measured by SPPB. However, when we observe the values for all measurement time points for this parameter, it becomes clear that we may have possibly suffered a ceiling effect for this measurement in our sample. The values for this measure indicate we had a high level of lower extremity functioning in our selected senior sample.

Although we had a significant change in the MoCA outcome measure, this finding should also be interpreted with prudence in relation to its clinical relevance. First, we had high values for this measure indicating high levels of cognitive functioning in our sample and, second, we know from previous research that we can only be confident that an observed change would not be due to measurement error when we see individuals changing their score with 4 or more points (Feeney et al., 2016) and this should be two points or above for group assessments (Krishnan et al., 2017). Based on these values it becomes clear that there was not enough room for meaningful improvement for this outcome in our sample.

### Limitations of the Study

A limitation of our study relates to the testing threat known to possibly occur in pre-post research design. It might have been that testing our subjects with for example the MoCA at pretest made some of the participants more aware of cognitive skills and, hence, “primed” these individuals for the test so that when they repeated the measurement they were ready for it in a way that they wouldn’t have been without the pretest. Mortality Threat, used metaphorically here, might have been another limitation. This means that people are dropping out of the study. It is difficult to estimate whether the observed loss of three individuals (±10%) between pretest and posttest was non-trivial notwithstanding that the reasons reported for dropping out were not related to the intervention. However, compared to rates that might be expected for community dwelling older trainees after 12 months (Nyman and Victor, 2012) the dropout rate seems acceptable and in line with what could be expected.

The used research design, although having strengths, also has some limitations attached to it. To deal with the single group threats to internal validity this study should be replicated using a more stringent research design in which a control group is considered; e.g., a randomized control design that also considers blinding of participants and assessors where possible. In this scenario, we would have two groups: one receiving the exergames and the other one doesn’t with the aim of ruling out the single-group threats to internal validity.

Overall we can conclude that the initiation of an exergame-based training in upright standing position indicates to improve neural drive to the lower extremities in older adults and seems an acceptable form of physical exercise for this group. Further studies with more stringent designs are needed to refute or confirm this finding.

## Data Availability Statement

The datasets generated for this study are available on request to the corresponding author.

## Ethics Statement

The ethics committee of the ETH Zurich, Switzerland (EK 2017-N-22) approved the study protocol. Before any measurements were performed, an informed consent according to the Declaration of Helsinki was administered and signed by each eligible participant.

## Author Contributions

EB and FG developed the research question. The concept and design were established by EB while FG acted as methodological council. NP and LR conducted the data acquisition, analysis, and interpretation of the results (secondary outcomes) with editing and improvement by EB and FG. FG performed EMG data analysis and interpretation of the EMG results which was edited and improved by EB. EB produced a first version of the manuscript. FG substantially revised the manuscript to bring it to its current version. All authors have read and approved the final manuscript.

## Conflict of Interest

EB was a co-founder of dividat, the spin-off company that developed the video step platform used for the training of the seniors and is associated to the company as an external advisor. No revenue was paid (or promised to be paid) directly to EB or his institution over the 36 months prior to submission of the work. The remaining authors declare that the research was conducted in the absence of any commercial or financial relationships that could be construed as a potential conflict of interest.

## Footnotes

1 https://www.daskehl.ch/
